# Tumor regression following intravenous administration of lactoferrin- and lactoferricin-bearing dendriplexes

**DOI:** 10.1016/j.nano.2015.04.006

**Published:** 2015-08

**Authors:** Li Ying Lim, Pei Yin Koh, Sukrut Somani, Majed Al Robaian, Reatul Karim, Yi Lyn Yean, Jennifer Mitchell, Rothwelle J. Tate, RuAngelie Edrada-Ebel, David R. Blatchford, Margaret Mullin, Christine Dufès

**Affiliations:** a Strathclyde Institute of Pharmacy and Biomedical Sciences, University of Strathclyde, Glasgow, United Kingdom; b College of Medical, Veterinary and Life Sciences, University of Glasgow, Glasgow, United Kingdom

**Keywords:** Cancer therapy, Gene delivery, Dendrimer, Lactoferrin, Lactoferricin

## Abstract

The possibility of using gene therapy for the treatment of cancer is limited by the lack of safe, intravenously administered delivery systems able to selectively deliver therapeutic genes to tumors. In this study, we investigated if the conjugation of the polypropylenimine dendrimer to lactoferrin and lactoferricin, whose receptors are overexpressed on cancer cells, could result in a selective gene delivery to tumors and a subsequently enhanced therapeutic efficacy. The conjugation of lactoferrin and lactoferricin to the dendrimer significantly increased the gene expression in the tumor while decreasing the non-specific gene expression in the liver. Consequently, the intravenous administration of the targeted dendriplexes encoding TNFα led to the complete suppression of 60% of A431 tumors and up to 50% of B16-F10 tumors over one month. The treatment was well tolerated by the animals. These results suggest that these novel lactoferrin- and lactoferricin-bearing dendrimers are promising gene delivery systems for cancer therapy.

**From the Clinical Editor:**

Specific targeting of cancer cells should enhance the delivery of chemotherapeutic agents. This is especially true for gene delivery. In this article, the authors utilized a dendrimer-based system and conjugated this with lactoferrin and lactoferricin to deliver anti-tumor genes. The positive findings in animal studies should provide the basis for further clinical studies.

## Graphical abstract

Tumor regression in a mouse A431 xenograft model after intravenous administration of lactoferrin- and lactoferricin-bearing diaminobutyric polypropylenimine (DAB) dendriplex encoding tumor necrosis factor (TNF) α.

**Figure:**
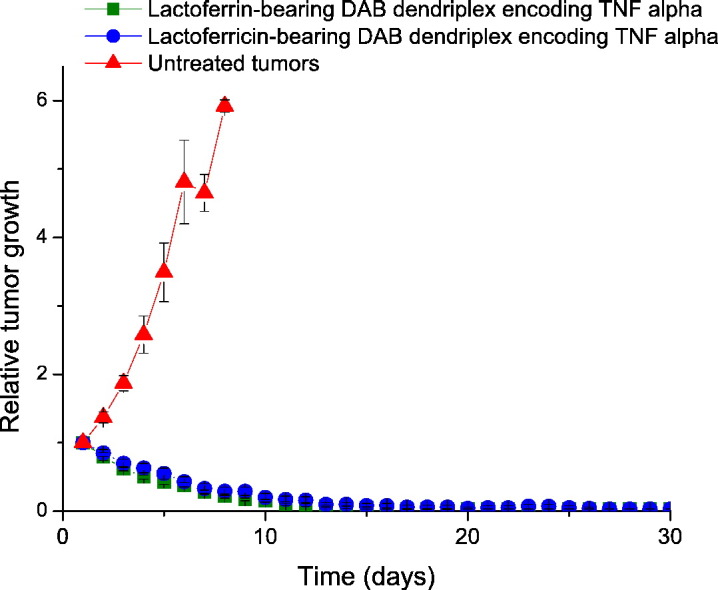

Despite numerous advances in the field of cancer gene therapy, the use of therapeutic genes in cancer treatment is still limited by the lack of safe, intravenously administered delivery systems able to carry therapeutic DNA selectively to the tumors, without secondary effects to healthy tissues.^1^

In order to remediate to this problem, numerous non-viral gene delivery systems are currently under development, due to advantages such as their low toxicity, stability and high flexibility regarding the size of the transgene delivered.^2,3^ Among these delivery systems, generation 3-diaminobutyric polypropylenimine dendrimer (DAB) appears to be particularly promising. We have recently demonstrated that the intravenous administration of this dendrimer conjugated to transferrin (Tf), whose receptors are overexpressed on cancer cells, resulted in gene expression mainly in the tumors after intravenous administration.^4^ Thus, DAB-Tf dendrimer complexed to a TNFα-encoding DNA led to a rapid and sustained tumor regression over one month, resulting in complete suppression of 90% of the tested A431 tumors and regression of the remaining 10%.^4^ Importantly, the treatment was well tolerated by the animals, with no apparent signs of toxicity.

Building on this study, we now would like to develop a novel gene-based therapeutic system with improved tumor targeting and therapeutic efficacy. To do so, we propose to replace the transferrin moiety by other promising tumor-targeting ligands of the same family that have been shown to have intrinsic anti-tumoral activity, such as lactoferrin and lactoferricin.

Lactoferrin (LF) and lactoferricin (LFC) are iron-binding members of the transferrin family, able to bind the transferrin receptors. In addition to their tumor delivery properties, these iron-carriers have recently been shown to have anti-cancer properties themselves, which make them highly attractive as part of a gene medicine.

LF has been shown to inhibit the proliferation of many cancer cell lines through induction of cell cycle arrest and modulation of the mitogen-activated protein kinase signaling pathway *in vitro*.^5^ The inhibition of tumor cell growth by LF may also be related to the ability of this protein to induce apoptosis of cancer cells by activating the Fas signaling pathway in cancerous cells.

Like LF, LFC has been shown to exert anti-tumor effects against a number of cancer cell lines. LFC is a potent inducer of apoptosis in various cancer types.^6^ LFC has also been reported to exert potent *in vivo* anti-tumor activity in mouse models of cancer. For example, direct injection of LFC into solid Meth A tumors causes tumor cell lysis and reduction in tumor size.^7^ In addition, subcutaneous administration of LFC inhibits tumor metastasis by metastatic murine L5178Y-ML25 lymphoma cells and B16-F10 melanoma cells.^8^ We therefore hypothesize that using LF and LFC as tumor-targeted ligands could improve the overall efficacy of the DAB delivery system.

The objectives of this study were therefore 1) to prepare and characterize lactoferrin- and lactoferricin-bearing DAB dendrimers and 2) to evaluate their targeting and therapeutic efficacy on cancer cells *in vitro* and *in vivo* after intravenous administration.

## Methods

### Cell lines and reagents

Lactoferrin and lactoferricin, generation 3-diaminobutyric polypropylenimine dendrimer (DAB) and the other chemicals were purchased from Sigma Aldrich (Poole, UK). The expression plasmids encoding Tumor necrosis factor (TNF) α (pORF9-mTNFα) and β-galactosidase (pCMVsport β-galactosidase) were obtained respectively from InvivoGen (San Diego, CA) and Invitrogen (Paisley, UK) and were purified using an Endotoxin-free Giga Plasmid Kit (Qiagen, Hilden, Germany). Passive lysis buffer was from Promega (Southampton, UK). Quanti-iT™ PicoGreen^®^ dsDNA reagent and tissue culture media were obtained from Invitrogen (Paisley, UK). Bioware^®^ B16-F10-luc-G5 mouse melanoma was obtained from Caliper Life Sciences (Hopkinton, MA). A431 human epidermoid carcinoma and T98G human glioblastoma were purchased from the European Collection of Cell Cultures (Salisbury, UK).

### Synthesis and characterization of lactoferrin- and lactoferricin- bearing DAB dendrimers

#### Conjugation of lactoferrin and lactoferricin to DAB

Lactoferrin (LF) and lactoferricin (LFC) were conjugated to generation 3- diaminobutyric polypropylenimine dendrimer (DAB) in a similar manner to that we previously reported for the preparation of other conjugates.^4,9–12^ DAB (24 mg) was added to lactoferrin or lactoferricin (6 mg) and dimethylsuberimidate (12 mg) in triethanolamine HCl buffer (pH 7.4, 2 mL). The coupling reaction was allowed to take place for 2 h at 25 °C whilst stirring. The conjugates were purified by size exclusion chromatography using a Sephadex G75 column and freeze-dried. The grafting of lactoferrin and lactoferricin to DAB was assessed by ^1^H NMR spectroscopy using a Jeol Oxford NMR AS 400 spectrometer.

#### Characterization of dendriplex formation

The ability of DNA to form complexes with DAB-LF and DAB-LFC dendrimers was assessed by PicoGreen^®^ assay, following the protocol provided by the supplier. PicoGreen^®^ reagent was diluted 200-fold in Tris-EDTA buffer (10 mM Tris, 1 mM EDTA, pH 7.5) on the day of the experiment. One milliliter of PicoGreen^®^ solution was added to 1 mL of dendrimer–DNA complexes prepared at various dendrimer:DNA weight ratios (20:1, 10:1, 5:1, 2:1, 1:1, 0.5:1, 0:1). The DNA concentration in the complexes (10 μg/mL) was kept constant during the experiment. The fluorescence intensity of the complexes was analyzed at various time points with a Varian Cary Eclipse Fluorescence spectrophotometer (Palo Alto, CA) (λ_exc_: 480 nm, λ_em_: 520 nm). Results were represented as percentage of DNA condensation and compared with those obtained for DAB-DNA complex (dendrimer:DNA weight ratio 5:1) (n = 4).

DNA condensation ability of DAB-LF and DAB-LFC was also assessed by agarose gel retardation assay (Supplementary data). Nanoparticles of DAB-LF and DAB-LFC complexed with DNA were also visualized by transmission electron microscopy^10^ (Supplementary data).

#### Dendriplex size and zeta potential measurement

Size and zeta potential of DAB-LF and DAB-LFC dendriplexes prepared at various dendrimer:DNA weight ratios (20:1, 10:1, 5:1, 2:1, 1:1, 0.5:1, 0:1) were measured by photon correlation spectroscopy and laser Doppler electrophoresis using a Zetasizer Nano-ZS (Malvern Instruments, Malvern, UK).

### *In vitro* biological characterization

#### Cell culture

A431, T98G and B16-F10-luc-G5 cell lines overexpressing Tf receptors were grown as monolayers in DMEM (for A431 and T98G cells) or RPMI-1640 medium (for B16-F10-luc-G5 cells) supplemented with 10% (v/v) fetal bovine serum, 1% (v/v) l-glutamine and 0.5% (v/v) penicillin–streptomycin. Cells were cultured at 37 °C in a humid atmosphere of 5% carbon dioxide.

#### *In vitro* transfection

Transfection efficacy of the DNA carried by DAB-LF and DAB-LFC dendrimers was assessed by a β-galactosidase transfection assay, using a plasmid DNA encoding β-galactosidase. A431, B16-F10 and T98G cells were seeded in quintuplicate at a density of 2 000 cells/well in 96-well plates. After 72 h incubation, the cells were treated with the DAB-LF and DAB-LFC dendriplexes at the following dendrimer:DNA weight ratios: 20:1, 10:1, 5:1, 2:1, 1:1, 0.5:1, 0:1. DNA concentration (10 μg/mL) was kept constant for all the formulations tested. Naked DNA served as a negative control; DAB-DNA (dendrimer:DNA weight ratio 5:1) served as a positive control. After 72 h incubation, cells were lysed with 1 × passive lysis buffer (50 μL/well) during 20 min. The cell lysates were then analyzed for β-galactosidase expression.^13^ Briefly, 50 μL of the assay buffer (2 mM magnesium chloride, 100 mM mercaptoethanol, 1.33 mg/mL ο-nitrophenol-β-galactopyranoside, 200 mM sodium phosphate buffer, pH 7.3) was added to each well containing the lysates. After 2 h incubation at 37 °C, the absorbance of the samples was read at 405 nm with a plate reader (Thermo Lab Systems, Multiscan Ascent, UK).

#### Cellular uptake

Imaging of the cellular uptake of the DNA carried by DAB-LF and DAB-LFC was carried out by confocal microscopy. Plasmid DNA encoding β-galactosidase was labeled with the fluorescent probe Cy3 using a Label IT® Cy3 Nucleic Acid Labeling kit, as described by the manufacturer. A431, B16-F10 and T98G cells were seeded on coverslips in 6-well plates (10^4^ cells/well) and grown at 37 °C for 24 h. They were then incubated for 24 h with Cy3-labeled DNA (2.5 μg DNA/well) complexed to DAB-LF, DAB-LFC and DAB (dendrimer:DNA weight ratios of 2:1 for DAB-LF and DAB-LFC, 5:1 for DAB).^11,14^ Control slides were treated with naked DNA. The cells were then washed three times with PBS and fixed with methanol for 10 min. DAPI was used to stain the nuclei and the cells were examined using a Leica TCS SP5 confocal microscope. DAPI was excited with the 405 nm laser line (bandwidth: 415-491 nm), whereas Cy3 was excited with the 543 nm laser line (bandwidth: 550-620 nm).

The mechanisms involved in the cellular uptake of DNA complexed to DAB-LF and DAB-LFC dendriplexes were investigated by treatment with uptake inhibitors and free Tf (Supplementary data).

#### *In vitro* anti-proliferative activity

Anti-proliferative activity of DAB-LF and DAB-LFC complexed with a TNFα expression plasmid was assessed in A431, B16-F10 and T98G cancer cell lines. The cells were seeded in quintuplicate at a density of 2000 cells/well in 96-well plates 72 h before treatment. Following seeding, they were incubated for 72 h with the DNA formulations at final concentrations of 1.28 × 10^− 3^ to 100 μg/mL. Anti-proliferative activity was evaluated by measuring the growth inhibitory concentration for 50% of the cell population (IC_50_) in an MTT assay. Absorbance was measured at 570 nm using a plate reader. Dose–response curves were fitted to percentage absorbance values to obtain IC_50_ values (three independent experiments, with n = 5 for each concentration level).

### *In vivo* study

The *in vivo* experiments described below were approved by the local ethics committee and performed in accordance with the UK Home Office regulations.

#### Biodistribution of gene expression

A431 cancer cells in exponential growth were subcutaneously implanted to both flanks of female immunodeficient BALB/c mice (1 × 10^6^ cells per flank). When tumors became palpable, vascularized and reached a diameter of 5 mm, the mice were treated with a single intravenous injection of DAB-LF, DAB-LFC and DAB dendrimers carrying β-galactosidase expression plasmid (50 μg of DNA). They were sacrificed 24 h after injection and their organs were removed, frozen in liquid nitrogen, before being analyzed.^13^

#### *In vivo* tumoricidal activity

A431 and B16-F10-luc-G5 cells were subcutaneously implanted to the mice as described above. The mice bearing vascularized, palpable tumors were treated by intravenous injection of DAB-LF and DAB-LFC dendrimers complexed with TNFα expression plasmid or with a non-therapeutic plasmid encoding β-galactosidase, the non-targeted DAB dendrimer carrying TNFα expression plasmid, and naked DNA (50 μg of DNA) once daily for 10 days. The weight of the mice was measured every day as a surrogate marker of toxicity and tumor volume was determined by caliper measurements (volume = d^3^ × π/6). Results were expressed as relative tumor volume and responses classified analogous to Response Evaluation Criteria in Solid Tumors (RECIST).^15^

### Statistical analysis

Results were expressed as means ± standard error of the mean. Statistical significance was assessed by one-way analysis of variance and Tukey multiple comparison post-test (Minitab^®^ software, State College, PE). Differences were considered statistically significant for P values lower than 0.05.

## Results

### Synthesis and characterization of lactoferrin- and lactoferricin- bearing DAB dendrimers

#### Conjugation of lactoferrin and lactoferricin to DAB

**Supplementary Figure 1:**
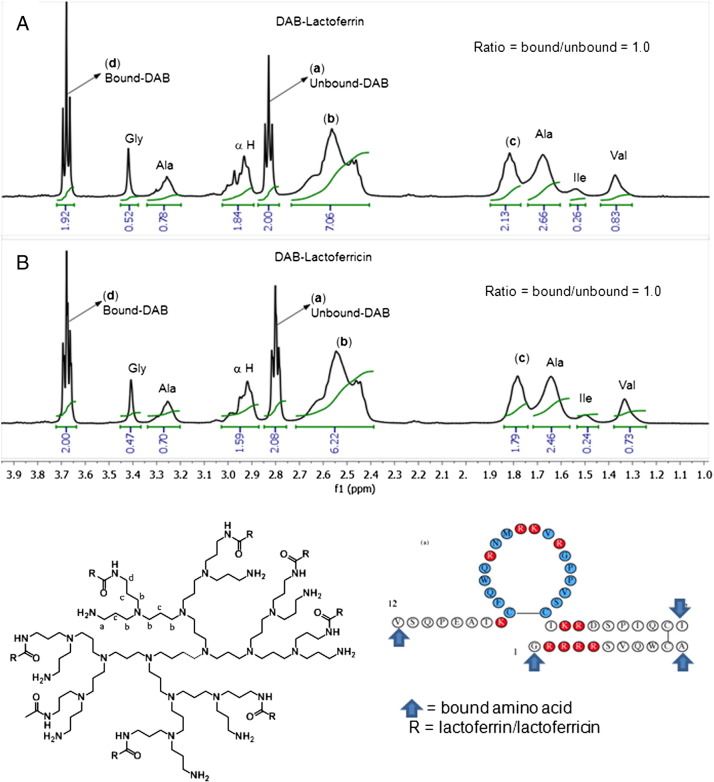
^1^H NMR spectra of DAB-Lactoferrin (DAB-LF) (A) and DAB-lactoferricin (DAB-LFC) (B).

The synthesis of DAB-LF and DAB-LFC was confirmed by ^1^H NMR (Supplementary Figure 1).

The synthesis of DAB-LF and DAB-LFC was confirmed by ^1^H NMR (Supplementary Figure 1).

#### Characterization of dendriplex formation

**Supplementary Figure 2:**
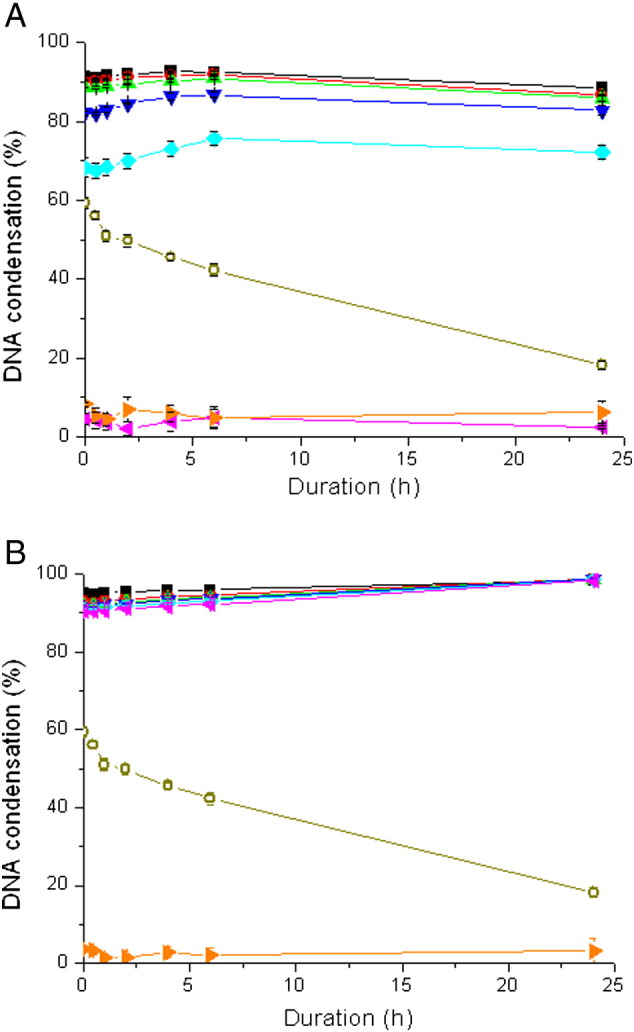
DNA condensation of DAB-LF (A) and DAB-LFC (B) dendriplexes using PicoGreen® reagent at various durations and dendrimer: DNA weight ratios : 20:1 (■, black), 10:1 (●, red), 5:1 (▲, green), 2:1 (▼, blue), 1:1 (♦, cyan), 0.5:1 (◄, pink), DNA only (►, orange) (empty symbol, dark yellow : DAB-DNA, dendrimer: DNA weight ratio: 5:1) . Results are expressed as mean ± SEM (n= 4).

DAB-LF and DAB-LFC were able to condense more than 80% and 90% of the DNA, respectively, at dendrimer:DNA weight ratios of 2:1 or higher (Supplementary Figure 2). DNA condensation occurred almost instantaneously and was found to be stable over at least 24 h. It increased with increasing weight ratios and was almost complete at a dendrimer:DNA weight ratio of 20:1 for DAB-LFC dendrimer. The DNA condensation observed for dendrimer:DNA weight ratios of 2:1 or higher was much higher than that observed for the unmodified dendrimer, which was of 60% at its best and decreasing with time.

DAB-LF and DAB-LFC were able to condense more than 80% and 90% of the DNA, respectively, at dendrimer:DNA weight ratios of 2:1 or higher (Supplementary Figure 2). DNA condensation occurred almost instantaneously and was found to be stable over at least 24 h. It increased with increasing weight ratios and was almost complete at a dendrimer:DNA weight ratio of 20:1 for DAB-LFC dendrimer. The DNA condensation observed for dendrimer:DNA weight ratios of 2:1 or higher was much higher than that observed for the unmodified dendrimer, which was of 60% at its best and decreasing with time.

**Supplementary Figure 3:**
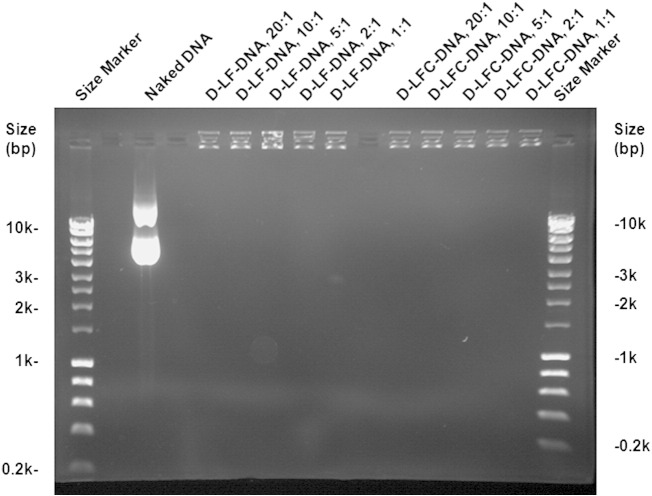
Gel retardation assay of DAB-LF and DAB-LFC dendriplexes at various dendrimer: DNA weight ratios: 20:1, 10:1, 5:1, 2:1, 1:1, and DNA only.

**Supplementary Figure 4:**
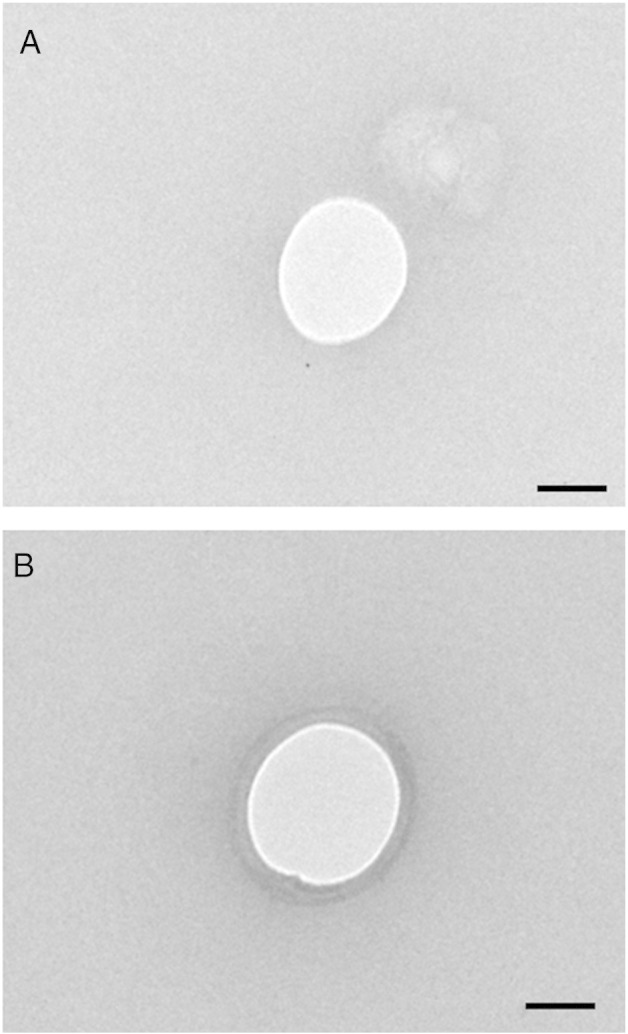
Transmission electron micrographs of DAB-LF (A) and DAB-LFC (B) dendriplexes (Bar: 100 nm).

A gel retardation assay confirmed the DNA condensation by DAB-LF and DAB-LFC dendrimers (Supplementary Figure 3). The formation of spherical nanoparticles of DAB-LF and DAB-LFC complexed to DNA was also demonstrated by electron microscopy (Supplementary Figure 4).

A gel retardation assay confirmed the DNA condensation by DAB-LF and DAB-LFC dendrimers (Supplementary Figure 3). The formation of spherical nanoparticles of DAB-LF and DAB-LFC complexed to DNA was also demonstrated by electron microscopy (Supplementary Figure 4).

#### Dendriplex size and zeta potential measurement

**Supplementary Figure 5:**
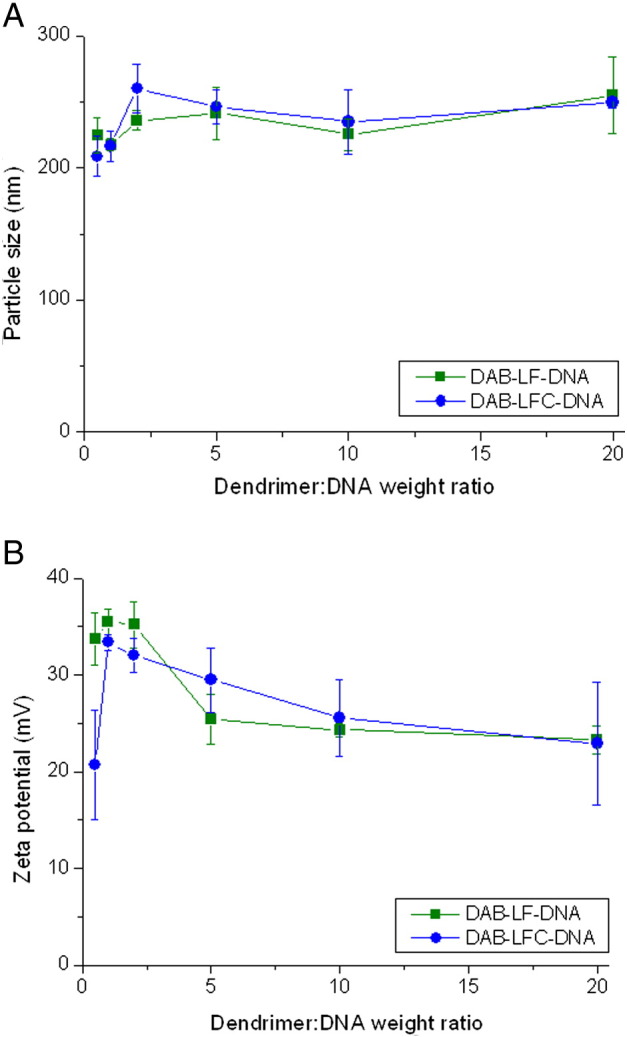
Size (A) and zeta potential (B) of DAB-LF and DAB-LFC dendriplexes at various dendrimer: DNA weight ratios: 20:1, 10:1, 5:1, 2:1, 1:1, 0.5:1. Results are expressed as mean ± SEM (n= 4).

DAB-LF and DAB-LFC dendriplexes displayed average sizes less than 300 nm, at all weight ratios tested (Supplementary Figure 5). The increase of dendrimer:DNA weight ratios did not have a significant impact on the dendriplexes size. Among the two tested targeted dendrimers, DAB-LF dendriplex at a dendrimer:DNA ratio of 2:1 was found to be the largest, with an average size of 260 ± 18 nm. In contrast, DAB-LF dendriplex at a dendrimer:DNA ratio of 0.5:1 was the smallest, with an average size of 208 ± 15 nm. The conjugation of LF and LFC to DAB led to an increase in the size of both DAB-LF and DAB-LFC dendriplexes compared to the unmodified DAB dendriplex, which had an average size of 196 nm (polydispersity index: 0.683).^10^

DAB-LF and DAB-LFC dendriplexes displayed average sizes less than 300 nm, at all weight ratios tested (Supplementary Figure 5). The increase of dendrimer:DNA weight ratios did not have a significant impact on the dendriplexes size. Among the two tested targeted dendrimers, DAB-LF dendriplex at a dendrimer:DNA ratio of 2:1 was found to be the largest, with an average size of 260 ± 18 nm. In contrast, DAB-LF dendriplex at a dendrimer:DNA ratio of 0.5:1 was the smallest, with an average size of 208 ± 15 nm. The conjugation of LF and LFC to DAB led to an increase in the size of both DAB-LF and DAB-LFC dendriplexes compared to the unmodified DAB dendriplex, which had an average size of 196 nm (polydispersity index: 0.683).^10^

Zeta potential experiments demonstrated that DAB-LF and DAB-LFC dendriplexes were bearing a positive surface charge at all dendrimer:DNA weight ratios. The zeta potential values of DAB-LF dendriplex reached their maximum (35 ± 2 mV) at a weight ratio of 2, before decreasing with increasing weight ratios and finally reaching their minimum (23 ± 1 mV) at a weight ratio of 20. The zeta potential values of DAB-LFC followed a similar pattern, namely reaching a maximum (33 ± 1 mV) at a weight ratio of 1 and then decreasing with increasing weight ratios to attain the same value as for DAB-LF dendriplex (23 ± 6 mV at a weight ratio of 20). The conjugation of LF and LFC to DAB increased the overall positive charge of the dendriplexes compared to non-targeted DAB-DNA (6 mV)^10^ for weight ratios over 2:1.

### *In vitro* biological characterization

#### *In vitro* transfection

The treatment of A431, B16-F10 and T98G cells with DAB-LF and DAB-LFC dendriplexes resulted in an increase in gene expression on all the tested cell lines for some dendrimer:DNA ratios.

**Figure 1:**
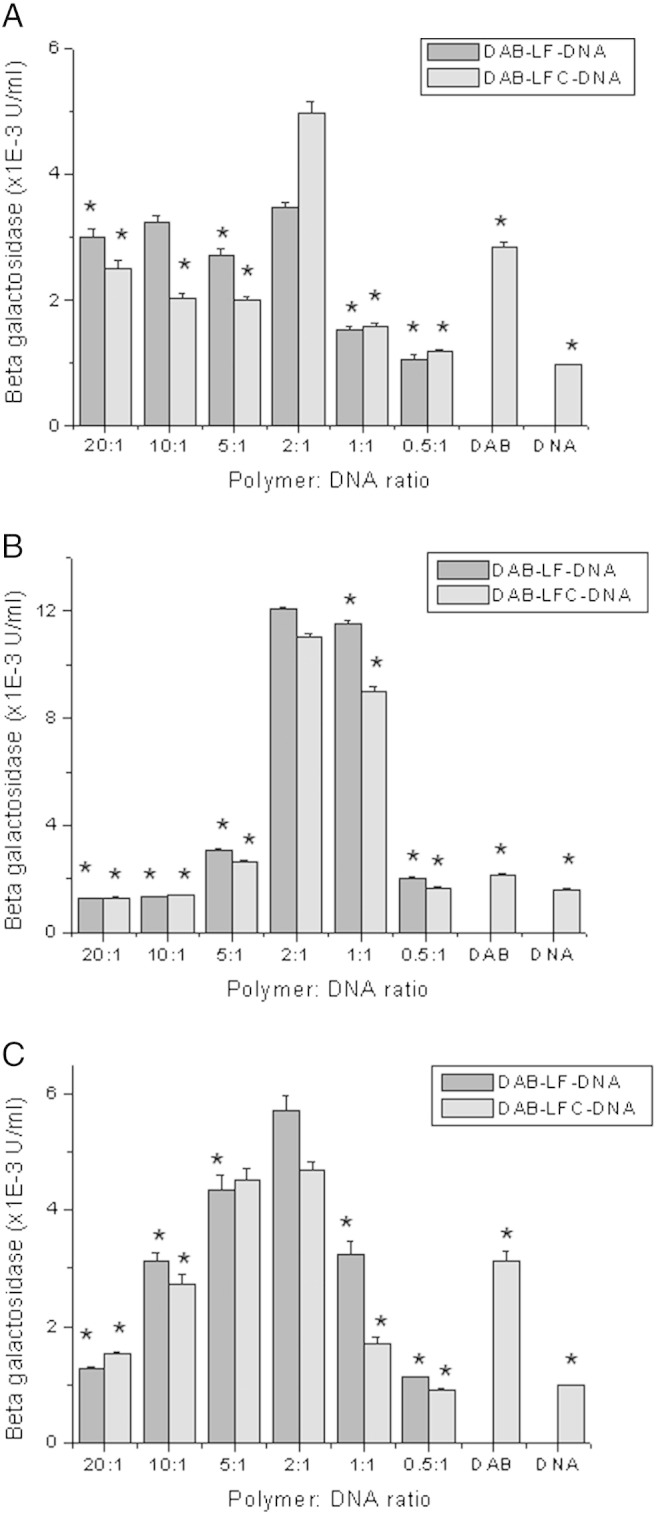
Transfection efficacy of DAB-LF and DAB-LFC dendriplexes at various dendrimer:DNA weight ratios in A431 **(A)**, B16-F10 **(B)** and T98G cells **(C)**. Results are expressed as the mean ± SEM of three replicates (n = 15). *P < 0.05 vs. the highest transfection ratio.

The highest transfection level after treatment with DAB-LF and DAB-LFC dendriplexes was obtained at a dendrimer:DNA weight ratio of 2:1 in A431, B16-F10 and T98G cells (Figure 1).

At this ratio, in A431 cells, treatment with DAB-LFC dendriplex led to the highest transfection (4.96 × 10^− 3^ ± 0.19 × 10^− 3^ U/mL), which was about 1.4-fold higher than that observed with DAB-LF dendriplex (3.45 × 10^− 3^ ± 0.10 × 10^− 3^ U/mL) (*P* < 0.001) (Figure 1, *A*).

By contrast, the highest transfection in B16-F10 cells was obtained after treatment with DAB-LF dendriplex (12.07 × 10^− 3^ ± 0.07 × 10^− 3^ U/mL and 11.01 × 10^− 3^ ± 0.12 × 10^− 3^ respectively for DAB-LF and DAB-LFC dendriplexes) (Figure 1, *B*).

In T98G cells as well, the highest transfection resulted from the treatment of the cells with DAB-LF dendriplex (5.71 × 10^− 3^ ± 0.24 × 10^− 3^ U/mL), which was about 1.2-fold higher than that of DAB-LFC dendriplex (4.67 × 10^− 3^ ± 0.16 × 10^− 3^ U/mL) (*P* < 0.01).

The conjugation of LF and LFC to DAB at their optimal dendrimer:DNA ratio led to an improved transfection compared to unconjugated DAB on all tested cell lines. Gene expression following treatment with DAB-LF dendriplex was respectively 1.2-fold, 5.6- fold and 1.8-fold higher than following treatment with DAB dendriplex on A431, B16-F10 and T98G cells (2.83 × 10^− 3^ ± 0.07 × 10^− 3^ U/mL on A431, 2.13 × 10^− 3^ ± 0.06 × 10^− 3^ U/mL on B16-F10, 3.12 × 10^− 3^ ± 0.17 × 10^− 3^ U/mL on T98G cells (*P* < 0.001)). Following treatment with DAB-LFC dendriplex, it was respectively 1.7-fold, 5.1-fold and 1.5-fold higher than that of DAB-DNA on A431, B16-F10 and T98G cells (*P* < 0.001).

#### Cellular uptake

**Figure 2:**
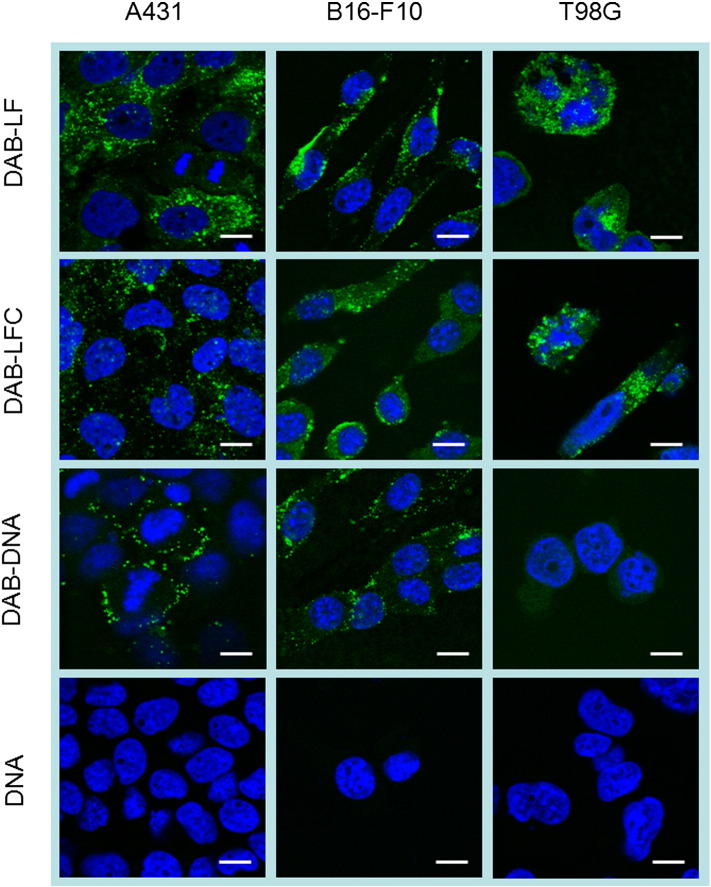
Confocal microscopy imaging of the cellular uptake of Cy3- labeled DNA (2.5 μg/well) either complexed with DAB-LF, DAB-LFC, DAB or in solution, after incubation for 24 h with A431 (left), B16-F10 (middle) and T98G cells (right). Blue: nuclei stained with DAPI (excitation: 405 nm laser line, bandwidth: 415-491 nm), green: Cy3-labeled DNA (excitation: 543 nm laser line. bandwidth: 550-620 nm) (Bar: 10 μm).

The cellular uptake of Cy3-labeled DNA carried by DAB-LF and DAB-LFC was qualitatively confirmed in the three cancer cell lines by confocal microscopy (Figure 2). Cy3-labeled DNA was disseminated in the cytoplasm after treatment with all DAB formulations in A431, B16-F10 and T98G cells. However, the DNA uptake appeared to be more pronounced in A431 and T98G cells treated with DAB-LF and DAB-LFC dendriplexes. B16-F10 cells treated with DAB-LFC dendriplex also appeared to be slightly more fluorescent than the cells treated with other DAB formulations. By contrast, cells treated with naked DNA did not show any Cy3-derived fluorescence.

**Supplementary Figure 6:**
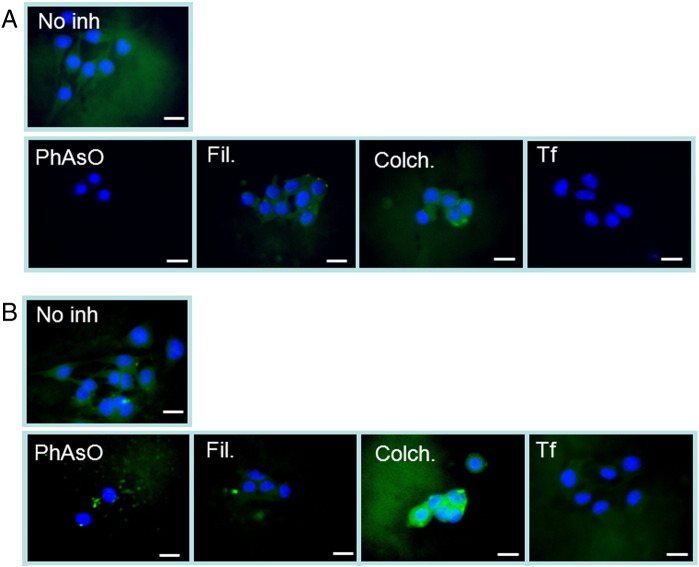
Epifluorescence microscopy imaging of the B16-F10 cellular uptake of Cy3- labeled DNA (2.5 µg/ well) complexed with DAB-LF (A) or DAB-LFC (B), following pre-treatment with various cellular uptake inhibitors: phenylarsine oxide (“PhAsO”), filipin (“Fil.”), colchicine (“Colch.”) and free Tf (“Tf”). (Blue: nuclei stained with DAPI, green: Cy3-labeled DNA) (Bar: 10 µm).

The cellular uptake of Cy3-labeled DNA complexed to DAB-LF was inhibited by phenylarsine oxide and free Tf, but not by filipin and colchicine (Supplementary Figure 6). By contrast, the uptake of DAB-LFC dendriplex was not inhibited by any of the inhibitors at the tested concentrations.

The cellular uptake of Cy3-labeled DNA complexed to DAB-LF was inhibited by phenylarsine oxide and free Tf, but not by filipin and colchicine (Supplementary Figure 6). By contrast, the uptake of DAB-LFC dendriplex was not inhibited by any of the inhibitors at the tested concentrations.

### *In vitro* anti-proliferative activity

The conjugation of LF and LFC to DAB led to a significant increase of *in vitro* anti-proliferative activity in the three tested cell lines. In A431 cells, the increase was respectively of 3.5-fold and 2.6-fold for DAB-LF and DAB-LFC dendriplexes compared to the unmodified DAB dendriplex (IC_50_ of 2.68 ± 0.63 μg/mL, 3.66 ± 0.22 μg/mL respectively for DAB-LF and DAB-LFC dendriplexes, 9.47 ± 1.15 μg/mL for unmodified DAB dendriplex) (Table 1). In B16-F10 cells, it was of 2.5-fold and 3.3-fold for DAB-LF and DAB-LFC dendriplexes compared to the unmodified DAB dendriplex (IC_50_ of 1.88 ± 0.15 μg/mL, 1.44 ± 0.25 μg/mL respectively for DAB-LF and DAB-LFC dendriplexes, 4.72 ± 0.32 μg/mL for unmodified DAB dendriplex). In T98G cells, however, the increase was at its highest, by 4.8-fold and 5.9-fold for DAB-LF and DAB-LFC dendriplexes compared to DAB dendriplex (IC_50_ of 6.20 ± 0.71 μg/mL, 5.01 ± 0.48 μg/mL respectively for DAB-LF and DAB-LFC dendriplexes, 29.84 ± 2.79 μg/mL for unmodified DAB dendriplex). By contrast, uncomplexed DAB-LF, DAB-LFC and naked DNA did not exert any cytotoxicity to the cells at the tested concentrations, thus raising the hypothesis that the conjugation of LF and LFC to DAB may hamper their intrinsic anti-cancer activity.

### *In vivo* study

#### Biodistribution of gene expression

**Figure 3:**
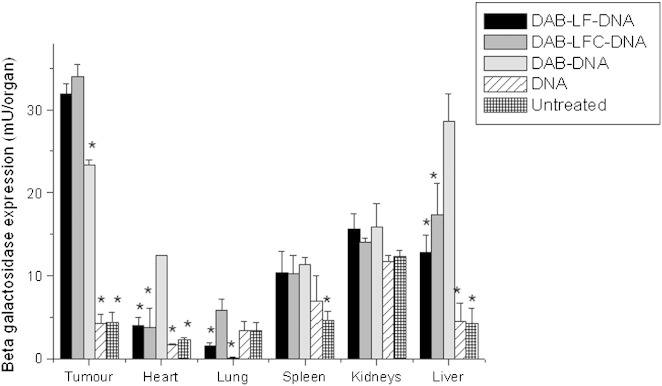
Biodistribution of gene expression after a single intravenous administration of DAB-LF, DAB-LFC and DAB dendriplexes (50 μg DNA administered). Results were expressed as milliunits β-galactosidase per organ (n = 5). *P < 0.05: highest gene expression treatment vs. other treatments for each organ.

The intravenous administration of control DAB dendriplex led to gene expression mainly in the liver (28.6 ± 3.3 mU β-galactosidase per organ) followed by the tumor (23.3 ± 0.5 mU β-galactosidase per organ) (Figure 3). By contrast, the conjugation of LF and LFC to DAB significantly increased by more than 1.3-fold the gene expression in the tumor (respectively 31.9 ± 1.2 and 33.9 ± 1.5 mU β-galactosidase in the tumor for DAB-LF and DAB-LFC dendriplexes (*P* < 0.001)), while decreasing the β-galactosidase amount in the liver by 2.2-fold following treatment with DAB-LF dendriplex (12.8 ± 2.1 mU β-galactosidase per organ, *P* < 0.001) and by 1.6-fold following treatment with DAB-LFC dendriplex (17.4 ± 3.7 mU β-galactosidase per organ, *P* < 0.001). The β-galactosidase amounts in the heart were also reduced to less than 5 mU β-galactosidase per organ. In the spleen and the kidneys, gene expression reached levels similar to those observed following treatment with non-conjugated DAB dendriplex.

#### *In vivo* tumoricidal activity

**Figure 4:**
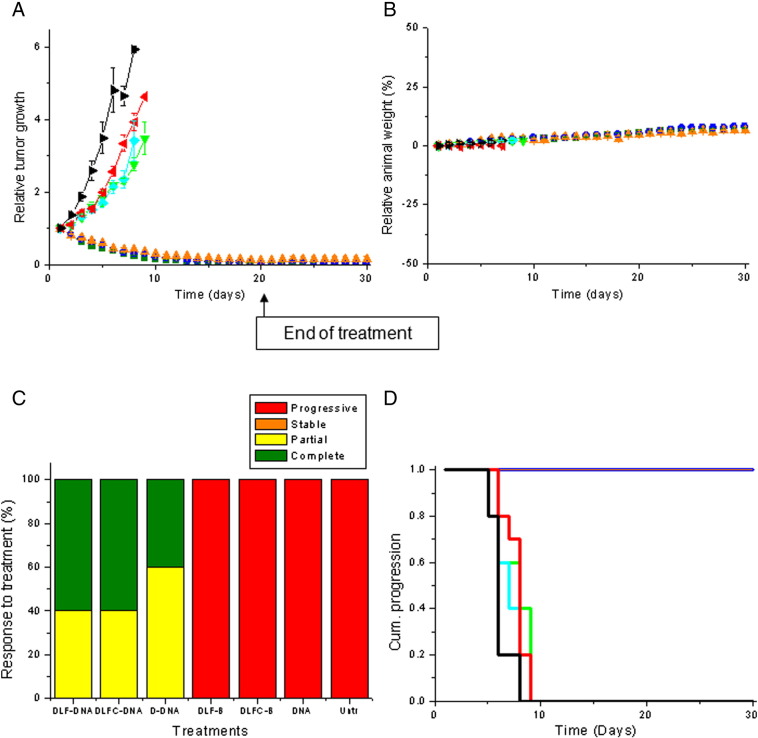
**(A)** Tumor growth studies in a mouse A431 xenograft model after intravenous administration of DAB-LF dendriplex carrying plasmid DNA encoding TNFα (50 μg/injection) (green), DAB-LFC dendriplex (blue), DAB dendriplex (orange), DAB-LF dendriplex carrying a non-therapeutic DNA encoding β-galactosidase (pale green), DAB-LFC dendriplex carrying a non-therapeutic DNA encoding β-galactosidase (pale blue), naked DNA (red) and untreated tumors (back) (n = 10). **(B)** Variations of the animal body weight throughout the treatment (Color coding as in **(A)**). **(C)** Overall tumor response to treatments at the end of the study. **(D)** Time to disease progression. The Y axis gives the proportion of surviving animals over time. Animals were removed from the study once their tumor reached 11 mm diameter (Color coding as in **(A)**).

The intravenous administration of DAB-LF, DAB-LFC and DAB complexed to TNFα expression plasmid resulted in tumor regression of A431 tumors (Figure 4, *A*). This effect was maintained for the whole duration of the experiment (30 days). By contrast, tumors treated with naked DNA or with the dendrimers complexed to a non-therapeutic DNA grew steadily at a growth rate close to that observed for untreated tumors.

**Figure 5:**
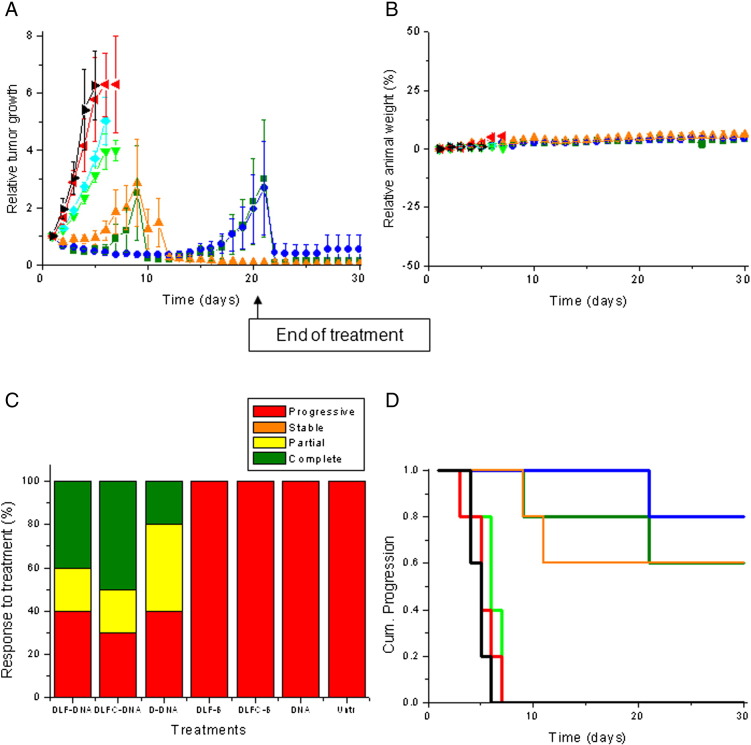
As in Figure 4 in a mouse B16-F10 model.

Treatment of the B16-F10 tumors with the 3 dendriplex formulations led to a different pattern, characterized by a high variability of response to treatment within a same group and an overall slowdown of tumor growth compared to naked DNA treatment (Figure 5, *A*).

No apparent signs of toxicity or weight loss were observed during the experiment, thus showing the good tolerability of the treatments by the mice (Figures 4, *B* and 5, *B*).

On the last day of the experiment, 60% of A431 tumors treated with DAB-LF and DAB-LFC dendriplexes had completely disappeared, which is an improvement compared to the 40% of A431 tumors disappearing following treatment with DAB dendriplex (Figure 4, *C*). The remaining A431 tumors treated by these 3 dendriplexes formulations showed a partial response.

Treatment of B16-F10 tumors with DAB-LF dendriplex led to 40% tumor disappearance and 20 % tumor regression (Figure 5, *C*). Replacing DAB-LF dendriplex by DAB-LFC dendriplex led to enhanced results, with 50% tumor disappearance and 20% tumor regression. These results were better compared to those obtained with control DAB dendriplex, which resulted in 20% tumor disappearance and 40% tumor regression. By contrast, all tumors treated with naked DNA, with the dendrimers complexed to a non-therapeutic DNA or left untreated were progressive for both tumor types.

This improved therapeutic effect resulted in an extended survival of 22 days compared to untreated mice, for all A431-bearing mice treated with targeted or control dendriplexes (Figure 4, *D*).

Sixty percent of B16-F10-bearing mice treated with DAB-LF and DAB-dendriplexes had their life extended by 24 days compared to untreated mice. This enhanced survival is similar that that observed following treatment with DAB-LFC dendriplex, but the percentage of surviving animals in that case increased to 80% (Figure 5, *D*). Treatment with naked DNA or with the dendrimers complexed to a non-therapeutic DNA did not extend the survival of the animals compared to untreated mice.

## Discussion

The use of gene therapy for the treatment of remote cancer and metastasis is limited by the inability of the therapeutic genes to specifically reach their target following intravenous administration, without secondary effects to healthy tissues. In order to overcome this issue, we hypothesized that the conjugation of DAB dendrimer to lactoferrin and lactoferricin, promising tumor-targeting ligands of the transferrin family that have intrinsic anti-tumoral activity and whose receptors are abundantly expressed on cancer cells, would improve the delivery of therapeutic DNA to cancer cells, resulting in better therapeutic efficacy *in vitro* and *in vivo*.

The conjugation of LF and LFC to DAB did not affect the ability of the dendrimer to complex DNA. An excess of dendrimer was however required to ensure efficient DNA condensation. Variations in the sensitivity of the nucleic acid stains used in the PicoGreen assay and the gel retardation assay could be responsible of the condensation discrepancy observed in these two assays for the dendriplexes at a dendrimer:DNA weight ratio of 1:1.

DAB-LF and DAB-LFC dendriplexes displayed sizes that should theoretically allow extravasation across tumor vasculature.^16^ They carried positive charges, higher than those of non-targeted DAB-DNA for weight ratios over 2:1. This zeta potential increase is most likely due to the presence of the positively charged amino acids of LF and LFC. It would eventually lead to an increase of the electrostatic interactions of the dendriplexes with negatively charged cellular membranes, resulting in an improved cellular uptake through internationalization mechanisms.^17^ DAB-LF and DAB-LFC therefore have the required physicochemical properties for being efficient gene delivery systems.

*In vitro*, transfection efficacy studies demonstrated that the conjugation of LF and LFC to DAB led to an enhanced transfection compared to unconjugated DAB on all the tested cell lines. The increased β-gal expression following treatment with DAB-LF and DAB-LFC at a dendrimer:DNA ratio of 2 most likely resulted from the higher cellular uptake of these dendriplexes at this ratio, as there is a strong correlation between cellular uptake and positive charge density of dendriplexes.^18^

The cellular uptake of Cy3-labeled DNA complexed to DAB-LF was inhibited by free Tf and by phenylarsine oxide, which is an inhibitor of clathrin-mediated endocytosis necessary for receptor-mediated endocytosis,^19^ but not by filipin and colchicine, both involved in non-specific endocytosis processes.^20,21^ These results therefore confirm the involvement of Tf receptor-mediated endocytosis in the cellular internalization of DNA complexed to DAB-LF.

Our cellular uptake results were in line with previous data obtained by Wei and colleagues,^22^ who demonstrated that the uptake of LF-conjugated, coumarin- and DiR-loaded liposomes was much higher than that of unconjugated liposomes in HepG2 human hepatoma cells. This outcome was also confirmed by Chen *et al*,^23^ who revealed that doxorubicin encapsulated in LF-bearing liposomes was more efficiently taken up by C6 glioma cells compared to other formulations. Our transfection results are in accordance with those obtained by Elfinger and colleagues in an experiment done with polyethylenimine (PEI) conjugated to LF.^24^ They demonstrated that LF-PEI polyplex exhibited a luciferase gene expression 5-fold higher than that of PEI polyplex in cells overexpressing LF receptors. Furthermore, we could not find any studies describing the transfection efficacy of LF-and LFC-bearing gene delivery systems in cancer cells to allow a comparison with our results. LF has been previously used as part of a gene therapeutic system against cancer, but as therapeutic LF cDNA instead of cancer-targeting moiety.^25–27^

The conjugation of LF and LFC to DAB increased the *in vitro* anti-proliferative activity of the dendriplex in the three tested cell lines. These results may be attributed to the improved transfection efficacy when treated with LF- and LFC-bearing DAB dendriplexes. DAB-LF and DAB-LFC dendriplexes were the most efficacious treatments on B16-F10 cells, probably as a result of their highest transfection efficacy on the same cell line. However, as for the transfection efficacy experiments, the lack of studies describing the anti-proliferative efficacy of LF- and LFC-bearing gene delivery systems in cancer cells prevented comparison with our results.

*In vivo*, DAB-LF and DAB-LFC dendriplexes administered intravenously resulted in an increased gene expression in subcutaneous tumors, while decreasing gene expression in the liver and the heart. Transferrin receptors are expressed in a range of cancer cells, but also on rapidly growing normal cells. The combination of active targeting, based on the use of ligands such as LF and LFC, and passive targeting, based on the accumulation of particulate delivery systems due to the enhanced permeability and retention,^28^ resulted in a tumor-selective targeting strategy. Similar improvements have been obtained by Wei *et al*^22^ when using LF-bearing PEGylated liposomes for hepatocellular carcinoma targeting. The authors demonstrated that the accumulation of DiR in tumors was significantly increased after the conjugation of LF to the PEGylated liposomes, whereas expression in the lungs and the other organs was reduced compared to the non-targeted liposomes.

The predominant gene expression in the tumor compared to the other organs is comparable to the gene expression pattern previously reported following intravenous administration of DAB-Tf dendriplex.^4^ However, when using Tf instead of LF and LFC as tumor targeting moieties, gene expression in the tumor was slightly higher (more than 35 mU/organ) than with LF or LFC. In addition, the β-galactosidase amounts in spleen, kidneys and liver were further decreased compared to those observed when using LF and LFC. DAB-LF and DAB-LFC therefore have the potential to deliver and express their carried DNA to remote tumors or metastases unsuitable for intratumoral treatments, but appear to be slightly less efficacious as DAB-Tf as tumor-targeting gene delivery systems.

This communication presents evidence that novel intravenously administered DAB-LF and DAB-LFC dendriplexes encoding TNFα led to tumor regression and even complete tumor suppression in some cases. In this study, DAB-LF and DAB-LFC have been shown to be able to increase the level of gene expression in tumors and the therapeutic efficacy compared to DAB dendriplex, resulting in complete tumor suppression of 40% of the A431 tumors and up to 50% of the B16-F10 tumors. Other researchers have already reported the ability of LF to target tumors *in vivo*,^22^ but did not assess the therapeutic efficacy of their delivery system yet. As far as we know, LF and LFC have been widely studied for their intrinsic anti-cancer properties, but have not been used so far as targeting moieties on a gene therapeutic system.

In the A431 xenograft model, the therapeutic effect of DAB-LF and DAB-LFC dendriplexes encoding TNFα was more pronounced than that obtained with B16-F10 tumors, contrarily to what was observed in our anti-proliferative assay *in vitro*. This could be explained by the fact that TNFα exerts its potent cytotoxic effects on tumors *in vivo* via the death receptor-dependent apoptotic pathway, but also via its anti-angiogenic effects, believed to be critical for its anti-cancer activity.^29^ It actually highlights the limitation of *in vitro* experiments for predicting the anti-cancer outcome of novel therapeutic systems *in vivo*.

In conclusion, we have demonstrated that novel intravenously administered lactoferrin- and lactoferricin-bearing DAB dendriplexes resulted in an improved tumor gene expression, while decreasing non-specific gene expression in the liver. Consequently, the intravenous administration of LF- and LFC-bearing, TNFα-encoding dendriplexes led to a sustained inhibition of tumor growth and even tumor suppression for 40% of the A431 tumors and up to 50% of the B16-F10 tumors, with long-term survival of the animals. In contrast, 100% of the tumors treated with naked DNA or left untreated were progressive. The animals did not show any signs of toxicity. These therapeutic effects, together with the lack of toxicity, potentially make lactoferrin- and lactoferricin-bearing DAB promising gene delivery systems for intravenous cancer therapy and should be further investigated to optimize their therapeutic potential.

## Figures and Tables

**Table 1:** Anti-proliferative efficacy of TNFα-encoding DNA complexed with DAB-LF, DAB-LFC and DAB in A431, B16-F10 and T98G cells, expressed as IC_50_ values (n = 15).

	IC_50_ (μg/mL) (mean ± SEM)		
Formulation	A431	B16F10	T98G
cplx DAB-LF	2.68 ± 0.63	1.88 ± 0.15	6.20 ± 0.71
cplx DAB-LFC	3.66 ± 0.22	1.44 ± 0.25	5.01 ± 0.48
cplx DAB	9.47 ± 1.15	4.72 ± 0.32	29.84 ± 2.79
DAB-LF only	> 100	> 100	> 100
DAB-LFC only	> 100	> 100	> 100
DAB only	> 100	> 100	> 100
DNA only	> 100	> 100	> 100
