# Supplementary material for: Tumor regression following intravenous administration of lactoferrin- and lactoferricin-bearing dendriplexes
Source: Nanomedicine. 2015 Aug;11(6):1445–54. doi: 10.1016/j.nano.2015.04.006 (PMC4509555; doi:10.1016/j.nano.2015.04.006)
Supplement: Supplementary file 1 — Supplementary material. [file mmc1.docx]

**METHODS (Supplementary data)**

***Synthesis and characterization of lactoferrin- and lactoferricin- bearing DAB dendrimers***

*Characterization of dendriplex formation*

DNA condensation ability of DAB-LF and DAB-LFC was also assessed by agarose gel retardation assay. Dendriplexes were prepared at a final DNA concentration of 20 µg/mL. After mixing with loading buffer, the samples (15 µL) were loaded on a 1X Tris-Borate-EDTA (TBE) (89 mM Tris base, 89 mM boric acid, 2 mM Na_2_-EDTA, pH 8.3) buffered 0.8% (w/v) agarose gel containing ethidium bromide (0.4 µg/mL), with 1x TBE as a running buffer and HyperLadder I as a DNA size marker. The gel was run at 50 V for 1 h and then photographed under UV light.

Nanoparticles of DAB-LF and DAB-LFC complexed with DNA were also visualized by transmission electron microscopy, as previously described. ^10^ Formvar/Carbon-coated 300 mesh nickel grids were glow discharged and specimens in distilled water were dried down with filter paper to a thin layer onto the hydrophilic support film. Twenty µL of 2% (v/v) aqueous methylamine vanadate stain (NanoVan pH8) was applied and the mixture dried down immediately using filter paper. Dried specimens were imaged with a LEO 912 energy filtering transmission electron microscope operating at 120 kV. Contrast enhanced, zero-loss energy filtered digital images were recorded with a 14 bit /2K CCD camera.

***In vitro biological characterization***

*Cellular uptake*

The mechanisms involved in the cellular uptake of DNA complexed to DAB-LF and DAB-LFC dendriplexes were investigated by treatment with uptake inhibitors and free Tf. B16-F10 cells were seeded and grown as described above. After removal of the medium, they were then pre-treated with phenylarsine oxide (10 µmol/L), filipin (5 µg/mL), colchicine (10 µmol/L) and free Tf (20 µmol/L) for 10 min at 37^o^C. The cells were then treated with Cy3-labeled DNA (2.5 μg/well) complexed to the dendrimers for 2h, before being washed and processed for observation using an E600FN Upright Epifluorescence microscope (Nikon, Tokyo, Japan).

**RESULTS (Supplementary data)**

***Synthesis and characterization of DAB-LF and DAB-LFC (Supplementary Figure 1)***

*Conjugation of lactoferrin and lactoferricin to DAB*

The synthesis of DAB-LF and DAB-LFC was confirmed by ^1^H NMR (Supplementary Fig. 1). Spin systems for each moiety were confirmed by ^1^H-^1^H COSY : ^1^H NMR (D_2_O) δ: DAB-(H_2_N-**C*H_2_***-CH_2_-CH_2_-N-) = 3.20-3.45; DAB (-N-**C*H_2_***-CH_2_-**C*H_2_***-N-) = 2.45-3.00; DAB (O=CHN-**CH_2_**-CH_2_) = 3.85-4.00; terminal Gly in lactoferrin/lactoferricin (1Hα) = 3.4; terminal Ala in lactoferrin/lactoferricin (1Hα) = 3.30; Ala (2Hβ) = 1.65; terminal Val in lactoferrin/lactoferricin (1Hα) = 2.94; Val (C*H_3_*) = 1.30; terminal Ile in lactoferrin/lactoferricin (1Hα) = 2.95; Ile (2Hβ) = 1.65 and 1.41 overlapping with Ala; Ile (2Hγ) = 1.75; Ile (2C*H_3_*) = 1.30 (overlapping).

The characteristic triplet peak for the C*H_2_* adjacent to peripheral primary amino group of DAB at δ 2.78 was shifted to 3.68 ppm in the NMR spectrum of a conjugated DAB- LF/LFC analogue. These results demonstrated that DAB has been successfully conjugated with the respective ligands. ^1^H NMR spectra showed that 50% of the surface group of dendrimer DAB was bound to 8 units of proteins as signified by the ratio of the integrals of resonances at ca. δ 3.69 and 2.78 for methylene units (**d** and **a**) attached to the amide-linked bound amino acid moiety and unbound free amine, respectively. Percentage conjugation of the proteins with the dendrimer was found to be 50% for both lactoferrin and lactoferricin, as previously observed when preparing amino acid- and transferrin-bearing DAB using the same simple one-step synthesis. ^4, 10^ Generation 3 diaminobutyric polypropylenimine dendrimer (DAB), with a MW of 1.7 kDa, had 16 units of terminal amine moiety. At 50% conjugation, 8 units of lactoferrin (83.1 kDa per unit) were bound to DAB, resulting to a MW of 667.7 kDa for DAB-LF. Eight units of lactoferricin (1.5 kDa per unit) bound to DAB yielded a MW of 13.7 kDa for DAB-LFC. Conjugation could occur at any of the terminal amino acids in lactoferrin and lactoferricin.

*Characterization of dendriplex formation*

A gel retardation assay confirmed the DNA condensation by DAB-LF and DAB-LFC dendrimers (Supplementary Fig. 3). At dendrimer: DNA weight ratios ranging from 20:1 to 1:1, DNA appeared to be fully condensed by DAB-LF and DAB-LFC, thus preventing ethidium bromide to intercalate with DNA. No free DNA was therefore visible at these ratios.

The formation of spherical nanoparticles of DAB-LF and DAB-LFC complexed to DNA was also demonstrated by transmission electron microscopy (Supplementary Fig. 4).
